# The predictive value of absolute lymphocyte counts on tumor progression and pseudoprogression in patients with glioblastoma

**DOI:** 10.1186/s12885-021-08004-2

**Published:** 2021-03-16

**Authors:** Jing Xi, Bilal Hassan, Ruth G. N. Katumba, Karam Khaddour, Akshay Govindan, Jingqin Luo, Jiayi Huang, Jian L. Campian

**Affiliations:** 1 Department of Medicine Division of Oncology, Washington University School of Medicine, 660 South Euclid Avenue, Campus Box 8056, St Louis, MO 63110 USA; 2 Department of Internal Medicine, Washington University School of Medicine, 666 S. Euclid Ave, Campus Box#8056, St. Louis, MO 63110 USA; 3 Trinity Health, 20555 Victor Parkway, Livonia, MI 48152 USA; 4 Department of Internal Medicine, Division of Hematology/Oncology, University of Illinois at Chicago, 820 S Wood Street (MC 675) Suite 100, Chicago, IL 60612 USA; 5 University College, Washington University in St. Louis, St. Louis, MO 63110 USA; 6 Washington University in St. Louis, One Brookings Drive, St. Louis, MO 63130 USA; 7 Department of Surgery and Siteman Cancer Center Biostatistics Core, Division of Public Health Sciences, Washington University School of Medicine, 666 S. Euclid Ave, Campus Box #8100, St. Louis, USA; 8 Department of Radiation Oncology, Washington University School of Medicine, 4921 Parkview Place, Campus Box #8224, St Louis, MO 63110 USA

**Keywords:** Absolute lymphocyte count, Pseudoprogression, Prognosis, Glioblastoma

## Abstract

**Background:**

Differentiating true glioblastoma multiforme (GBM) from pseudoprogression (PsP) remains a challenge with current standard magnetic resonance imaging (MRI). The objective of this study was to explore whether patients’ absolute lymphocyte count (ALC) levels can be utilized to predict true tumor progression and PsP.

**Methods:**

Patients were considered eligible for the study if they had 1) GBM diagnosis, 2) a series of blood cell counts and clinical follow-ups, and 3) tumor progression documented by both MRI and pathology. Data analysis results include descriptive statistics, median (IQR) for continuous variables and count (%) for categorical variables, *p* values from Wilcoxon rank sum test or Fisher’s exact test for comparison, respectively, and Kaplan-Meier analysis for overall survival (OS). OS was defined as the time from patients’ second surgery to their time of death or last follow up if patients were still alive.

**Results:**

78 patients were included in this study. The median age was 56 years. Median ALC dropped 34.5% from baseline 1400 cells/mm^3^ to 917 cells/mm^3^ after completion of radiation therapy (RT) and temozolomide (TMZ). All study patients had undergone surgical biopsy upon MRI-documented progression. 37 had true tumor progression (47.44%) and 41 had pseudoprogression (52.56%). ALC before RT/TMZ, post RT/TMZ and at the time of MRI-documented progression did not show significant difference between patients with true progression and PsP. Although not statistically significant, this study found that patients with true progression had worse OS compared to those with PsP (Hazard Ratio [HR] 1.44, 95% CI 0.86–2.43, *P* = 0.178). This study also found that patients with high ALC (dichotomized by median) post-radiation had longer OS.

**Conclusion:**

Our results indicate that ALC level in GBM patients before or after treatment does not have predictive value for true disease progression or pseudoprogression. Patients with true progression had worse OS compared to those who had pseudoprogression. A larger sample size that includes CD4 cell counts may be needed to evaluate the PsP predictive value of peripheral blood biomarkers.

## Background

Gliomas are the most common form of central nervous system (CNS) malignant tumors in adults [1]. Current research in the United States (U.S) shows that six cases of gliomas are diagnosed per 100,000 people every year [2]. Glioblastomas (GBM), an aggressive form of high-grade gliomas (HGG), make up 16% of all primary brain tumors [3]. The current standard treatment for patients with HGG includes surgery followed by three lymphotoxic therapies: temozolomide (TMZ), radiation (RT), and glucocorticoids. These three treatments result in toxicity to lymphocytes and immunosuppressive effects in HGG patients contributing to death.

Previous literature has demonstrated that the immune system plays a major role in suppressing the development and growth of primary brain tumors [4–7]. Patients with pretreatment lymphopenia were found to have poor clinical outcomes. Furthermore, treatment-related lymphopenia (TRL) has been correlated with inferior overall survival (OS) due to tumor progression [6]. Current standard treatment regimens for brain tumors deliver lymphotoxic radiation doses to 99% of circulating blood resulting in significant lymphopenia and consequently shorter overall survival [8]. Partial brain RT may independently result in systemic lymphopenia and this is further aggravated by chemotherapy. TRL in patients with malignant gliomas is common and is also associated with significant adverse clinical outcomes [5]. Two previous studies showed that GBM patients receiving concurrent chemoradiation, which may result in TRL, had shorter OS [6, 7]. Similar findings were reported in patients with pancreatic adenocarcinoma [4, 9, 10], and stage III non-small cell lung cancer (NSCLC) [4, 11]. TRL was also found to be associated with early disease progression in patients with newly diagnosed squamous head and neck cancer (HNSCC) [12].

Evaluation of post-treatment glioblastoma presents numerous challenges, including pseudoprogression (PsP) – also known as treatment effect, which appears on magnetic resonance imaging (MRI) as an enhancing lesion weeks to months after radiotherapy [13–15]. By mimicking tumor progression without actual clinical deterioration, PsP affects a substantial number of glioblastoma patients. PsP most likely stems from a temporary pause in myelin biosynthesis due to oligodendrocyte injury or local inflammatory reactions as a result of treatment [14]. Distinguishing true tumor progression from PsP is essential for the evaluation of patient outcomes. Current techniques to characterize PsP, such as MRI and positron emission tomography (PET), lack strong criteria for differentiating post-treatment effects from actual tumor progression [14]. Additionally, these methods, along with invasive techniques such as tissue biopsy and resection, are expensive, labor-intensive, and pose serious risks to patients [16].

Circulating glioma biomarkers are a novel modality that have been actively investigated recently as a potential tool to augment the differentiation between true tumor progression and the PsP in glioma. Several classes of biomarkers including angiogenesis, inflammation related proteins, circulating tumor cells (CTCs) and immune cells have been studied [17]. CD4+ and CD8+ tumor infiltrating lymphocytes (TILs) have been well documented in gliomas as a predictor of post-treatment patient outcomes and high percentages of CD4+ and CD8+ TILs have been correlated with the presence of PsP [18]. In addition, it has been reported that the levels of peripheral blood CD4 cell counts are associated with longer survival in patient with GBM [6]. However, CD4 cell counts are not routinely tested. On the contrary, ALC is often available from routine CBC testing and it can be utilized as a surrogate for CD4 cell counts. Low ALC post chemoradiation has been associated with reduced survival in elderly patients with GBM [19]. Based on prior promising research findings. This retrospective study was designed to investigate whether ALC could potentially be a useful biomarker for augmentation of response assessment in glioma. This study hypothesizes that high ALC will correlate with PsP in GBM patients.

## Methods

### Patient population

The study was reviewed and granted approval by the Institutional Review Board (IRB) of Washington University in St. Louis. Patients were identified retrospectively through a collaborative database of GBM patients at Washington University School of Medicine in St. Louis and Barnes-Jewish Hospital. The required eligibility criteria included (1) ≥ 18 years of age, (2) GBM initial diagnosis between 2010 and 2018, (3) a series of cell blood counts, (4) with clinical follow-up monitored, (5) KPS score ≥ 70, and (6) tumor progression status (absence/presence of tumor) documented by both MRI and pathology. A total of 78 patients were identified as eligible and were included in the analysis.

### Treatment and total lymphocyte count examination

Information related to prognostic factors in glioblastoma multiforme (WHO grade IV) was obtained from each patient’s medical record. GBM prognostic factors included MGMT methylation status, KPS, and extent of surgical resection. The degree of surgical resection was defined as gross total resection, subtotal resection, or biopsy. Additional data collected included age at diagnosis, sex, race, prior radiotherapy, prior chemotherapy, prior immunotherapy, baseline steroid use, anticonvulsant use, radiotherapy fractions, adjuvant chemotherapy, presence of infection, as well as glioma histopathology, tumor grade, tumor size, tumor location, second surgery histopathology, and ALC with CD4 counts at specific time points where available. The variables were dichotomized or further categorized as necessary. Baseline steroid use was defined as any glucocorticoid therapy use before the initiation of treatment. ALC data was collected along with CD4 counts (if available) at baseline before beginning chemoradiation, after chemoradiation, and at the time of MRI-demonstrated tumor progression. Surgical samples from the secondary surgery for each patient were subject to pathological examination to confirm true or PsP. PsP or treatment effect was largely characterized by evidence of significant tissue necrosis without active tumor growth whereas documented mixed progression involved histopathological residual tumor and necrotic cells. For purposes of comparative analyses, both necrosis and mixed progression were categorized as pseudoprogression (non-tumor). Overall survival was calculated from the date of second surgery until the date of death or censored at the date of the last clinic follow-up if patients were still alive.

### Statistical analysis

Patient baseline characteristics were summarized using descriptive statistics, and the difference between lymphopenia groups were compared using Chi-square test, Fisher’s exact test, Wilcoxon rank sum test as appropriate. Wilcoxon rank sum test was used to compare ALC between true progression and pseudoprogression at each time point. ROC curves of ALC at each time point were generated for true progression versus PsP. ALC was analyzed in binary scale as dichotomized by 1. Empirical survival probability was estimated using the Kaplan-Meier product limit method and survival difference between groups was compared using log-rank test. Hazard ratio was estimated from Cox proportional hazard model, accompanied with 95% confidence interval. All analyses were two-sided and significance was set at an alpha level of 5%. Statistical analysis was performed using R (version 3.1, https://cran.r-project.org/).

## Results

Seventy eight adult patients were included in the study analysis. The median age at diagnosis for patients in this cohort was 56 (range = 28–72), half of the study patients were male, 94.87% were white (*n* = 74), and median KPS at baseline was 85 (range = 80–90). Patients who were found to have PsP after their second surgery had higher baseline median KPS (median = 90, range = 80–90) compared to those who had true progression after their second surgery (median = 80, range = 70–90). This difference was not statistically significant. Furthermore, 30% of patients (*n* = 21) had detected MGMT (O^6^-methylguanine–DNA methyltransferase) DNA-repair gene and 71.43% of patients (*n* = 55) had undergone a gross total resection.

More than 97% of study patients had received concurrent chemoradiation and adjuvant chemotherapy, 50% of study patients were using steroids at baseline (*n* = 38) and 97.44% of patients had had no prior immunosuppression treatment. Only 5.13 and 2.56% of study patients had had prior RT (*n* = 4) and prior chemotherapy (n = 2), respectively. Pathology results from the second surgery (to confirm MRI-documented progression) revealed that 47.44% of patients (*n* = 37) had true tumor progression, and 41 had PsP (52.56%), including 29 who had mixed treatment effect and residual tumor (37.18%), and 12 who had necrosis (15.38%). At the time of study analysis, 59 out of 78 study patients (75.64%) had died. Between patients with true progression and PsP, there were no significant differences in demographic data, histology, MGMT methylation status, KPS, prior chemotherapy, prior immunosuppression therapy, baseline steroid use, treatment regimen (concurrent RT/TMZ versus RT only), adjuvant chemotherapy, presence of infection at baseline and laboratory data (baseline ALC and CD4 count).

Baseline demographic and clinical information for this cohort of patients has been summarized in Table 1.

**Table 1:** Patient demographics, clinical data and lab lymphocytes summary, by all and by pathology (tumor vs. non-tumor) at the second surgery

	All Patients (***n*** = 78)	Non-Tumor (***n*** = 41)	Tumor (n = 37)	***P***-value
**Demographics**				
**Age at diagnosis: median (range)**	56 (44.25–63.5)	59 (51–65)	53 (38–60)	0.015994
**Male: No. (%)**	39 (50)	20 (48.78)	19 (51.35)	
**Race: No. (%)**				0.046301
White, non-Hispanic	74 (94.87)	41 (100)	33 (89.19)	
Black/African American, non-Hispanic	4 (5.13)	0 (0)	4 (10.81)	
**Clinical Data**				
**Histology**				0.652495
Glioblastoma multiforme: No. (%)	74(94.87)	39(95.12)	35(94.59)	
Glioblastoma small cell variant: No. (%)	1(1.28)	1(2.44)	0(0)	
Glioblastoma with oligodendroglioma: No. (%)	1(1.28)	1(2.44)	0(0)	
Glioblastoma, small cell variant: No. (%)	1(1.28)	0(0)	1(2.7)	
High grade glioneuronal tumor: No. (%)	1(1.28)	0(0)	1(2.7)	
**Extent of surgical resection (*N*** **= 77)**				0.040166
biopsy	5(6.49)	0(0)	5(13.51)	
GTR	55(71.43)	32(80)	23(62.16)	
subtotal	17(22.08)	8(20)	9(24.32)	
**MGMT Methylation Status (*N*** **= 70)**				1
Methylated: No. (%)	21(30)	11(28.95)	10(31.25)	
Unmethylated: No. (%)	49(70)	27(71.05)	22(68.75)	
**KPS**	85(80 ~ 90)	90(80 ~ 90)	80(70 ~ 90)	0.100858
**Prior RT**				0.046301
No	74(94.87)	41(100)	33(89.19)	
Yes	4(5.13)	0(0)	4(10.81)	
**Prior chemo**				0.221778
No	76(97.44)	41(100)	35(94.59)	
Yes	2(2.56)	0(0)	2(5.41)	
**Prior immune suppression tx**				1
No	76(97.44)	40(97.56)	36(97.3)	
Yes	2(2.56)	1(2.44)	1(2.7)	
**Baseline steriod use (*N*** **= 76)**				1
No	38(50)	21(51.22)	17(48.57)	
Yes	38(50)	20(48.78)	18(51.43)	
**Treatment regimen**				0.494838
Concurrent RT/TMZ	76(97.44)	39(95.12)	37(100)	
RTonly	2(2.56)	2(4.88)	0(0)	
**Adjuvant Chemo**				1
No	1(1.28)	1(2.44)	0(0)	
Yes	77(98.72)	40(97.56)	37(100)	
**Infection**				0.702159
No	71(91.03)	38(92.68)	33(89.19)	
Yes	7(8.97)	3(7.32)	4(10.81)	
**Pathology at 2nd Surgery**				0.000000
mixed	29(37.18)	29(70.73)	0(0)	
necrosis/tx effect	12(15.38)	12(29.27)	0(0)	
tumor	37(47.44)	0(0)	37(100)	
**Laboratory Data**				
**ALC_beforeRT (*N*** **= 75):** median (range)	1.4 (0.9–1.9)	1.2 (0.9–1.9)	1.4 (0.9–2.3)	0.303656
**ALC_postRT (*N*** **= 73):** median (range)	0.9 (0.6–1.3)	1 (0.6–1.3)	0.9 (0.68–1.23)	0.742197
**ALC_MRIProgression (N = 74):** median	1 (0.7–1.5)	1 (0.72–1.5)	0.95 (0.7–1.62)	0.777925
**Baseline CD4 counts (*N*** **= 14):** median (range)	908 (619–1070.5)	1054 (888.25–1172.5)	763.5 (593.25–950)	0.217997
**POST RT/Chemo CD4 count (*N*** **= 23):** median (range)	344 (269.5–490.5)	404.5 (276.75–530.25)	344 (263–345)	0.377232
**CD4 count_MRIProgression (*N*** **= 15):** median (range)	340 (253–532)	484.5 (321–705.25)	253 (175–299.5)	0.048736

**Fig. 1:**
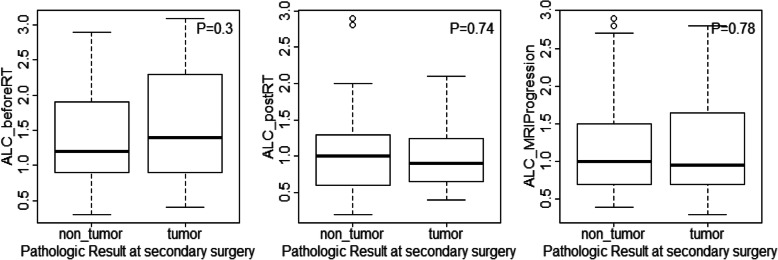
Boxplot of Absolute lymphocyte count (ALC) before concurrent radiation therapy and temozolomide (RT/TMZ), after RT/TMZ, and at the first MRI documented progression

**Fig. 2:**
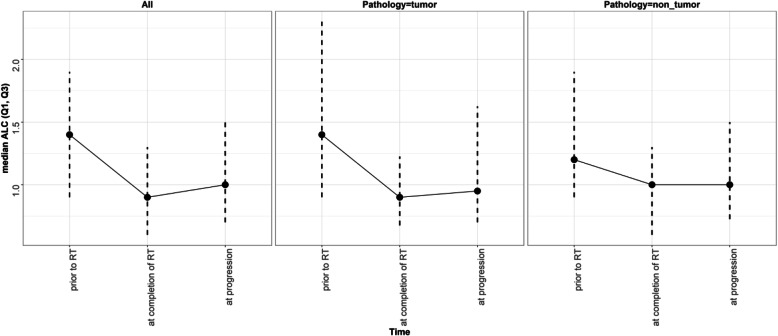
ALC Median Curve

The baseline median ALC for all patients before chemoradiation was 1400 cells/mm^3^ (900–1900 cells/mm^3^). After the completion of RT/TMZ, ALC dropped to a median of 900 cells/mm^3^ (600–1300 cells/mm^3^). Median ALC when MRI documented progression was 1000 cells/mm^3^ (700–1500 cells/mm^3^), slightly increased from the post radiation ALC. ALC before RT/TMZ, post RT/TMZ and at the time of MRI-demonstrated progression (confirmed by second surgical biopsy) did not differ significantly between the patients with true progression and pseudoprogression (**See** Figs. 1 and 2). Study results demonstrated significant differences between these two groups of patients in extent of surgical resection, prior RT, and pathology after their second surgery (to confirm true progression vs. pseudoprogression).

**Fig. 3:**
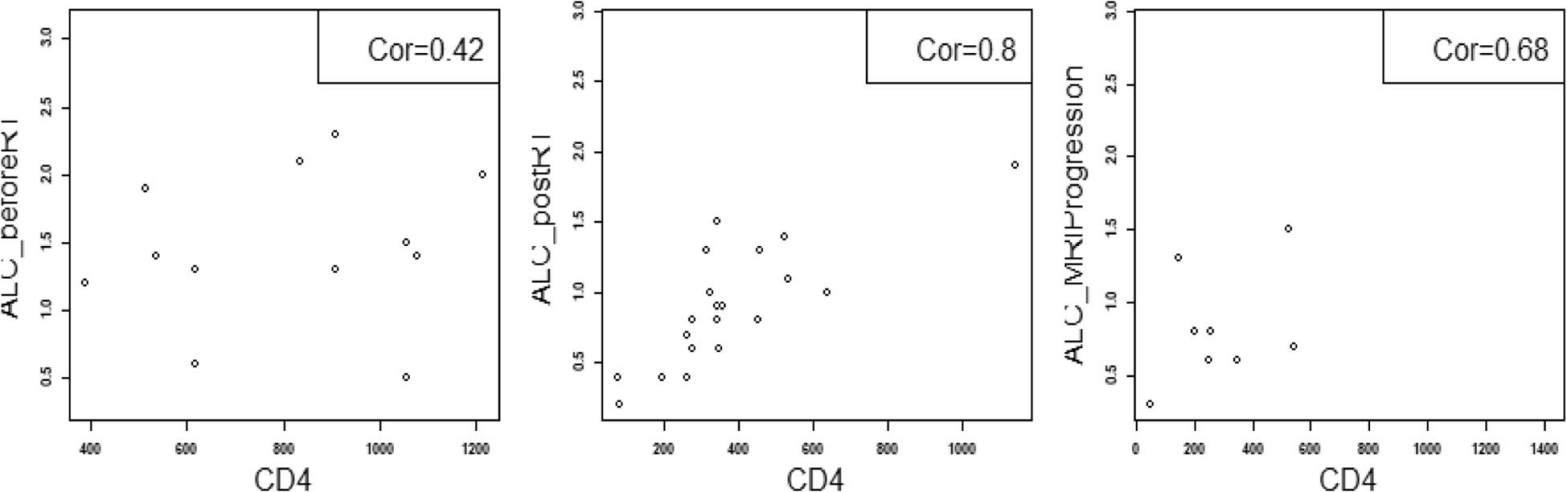
CD4 and ALC at the same time point are correlated

**Fig. 4:**
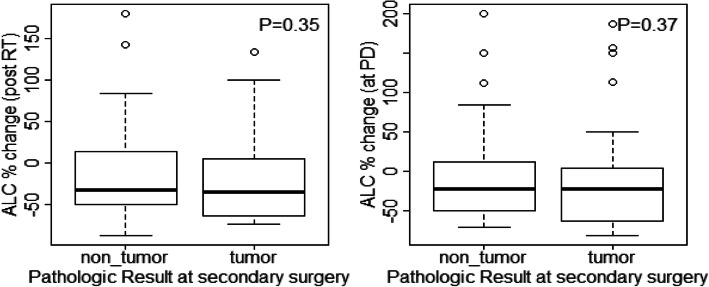
ALC % change post RT and at progression from prior to RT

**Fig. 5:**
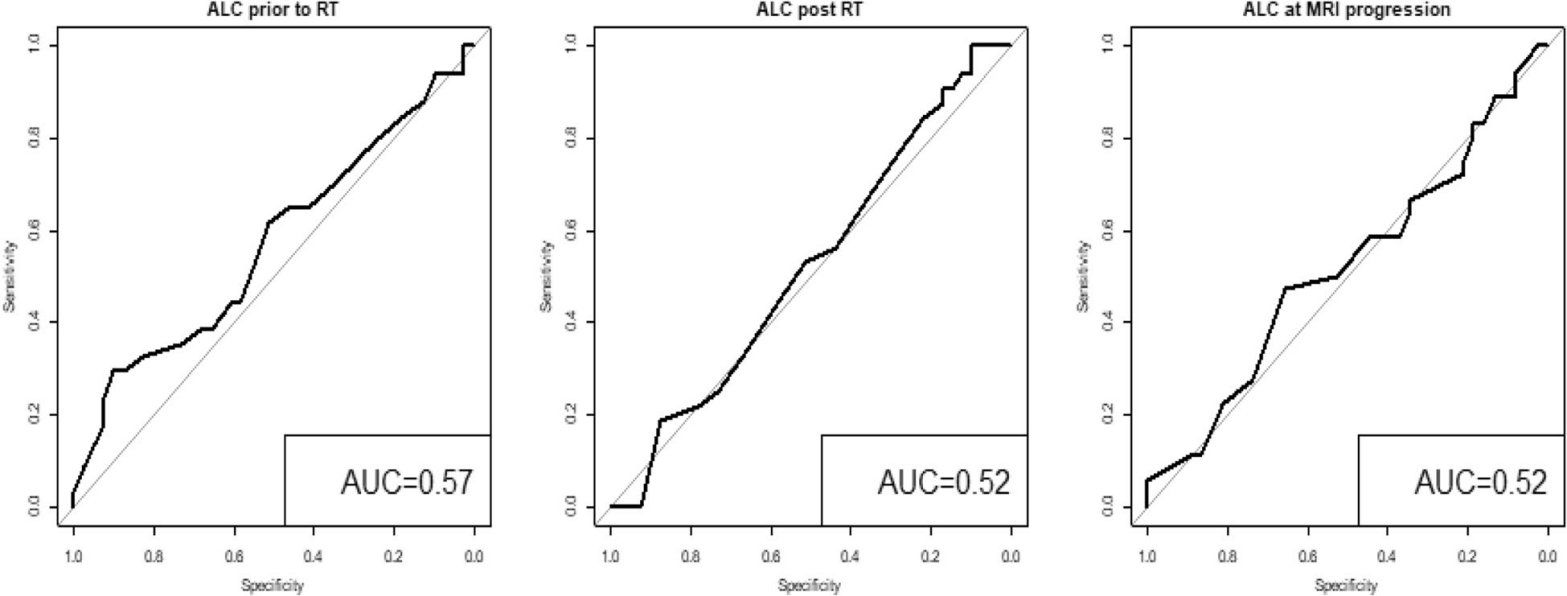
ROC analysis of ALC % change for tumor/non tumor

**Fig. 6:**
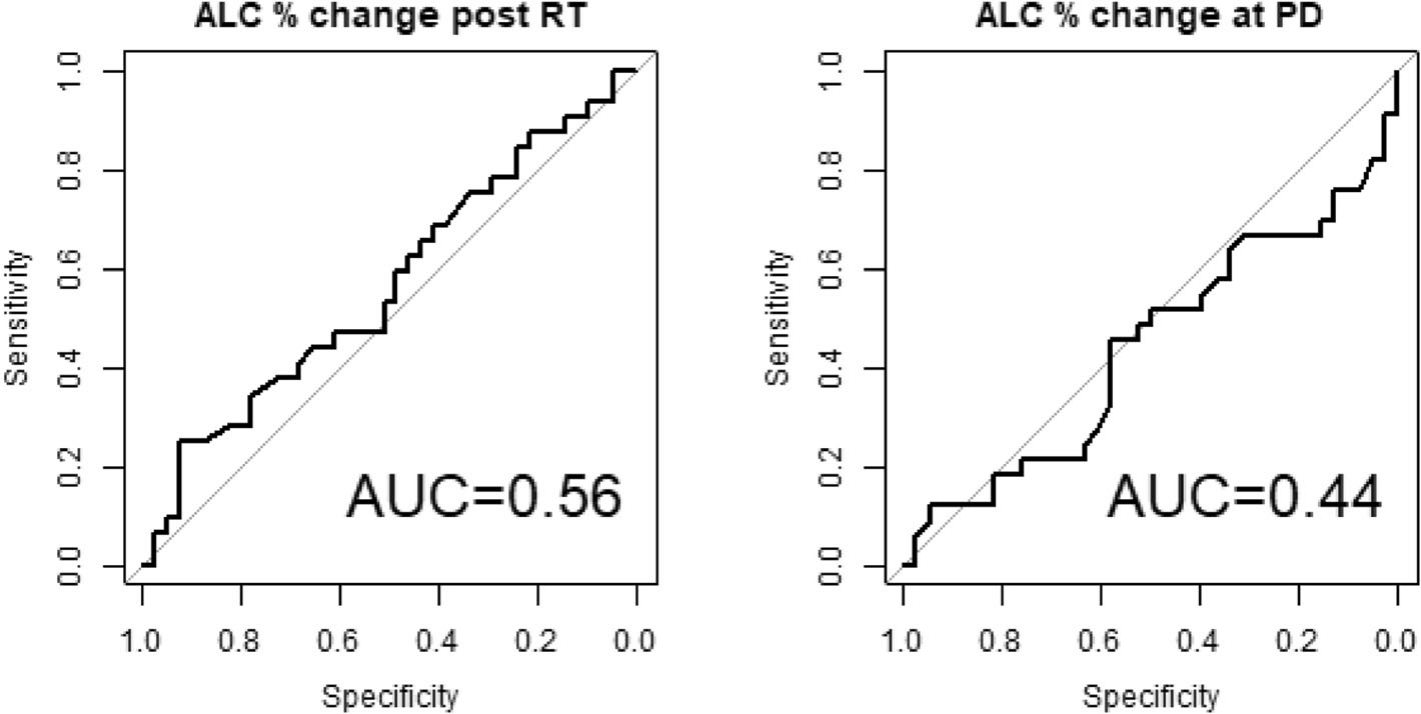
ROC analysis of each of the ALC at before, post RT and at progression

**Fig. 7:**
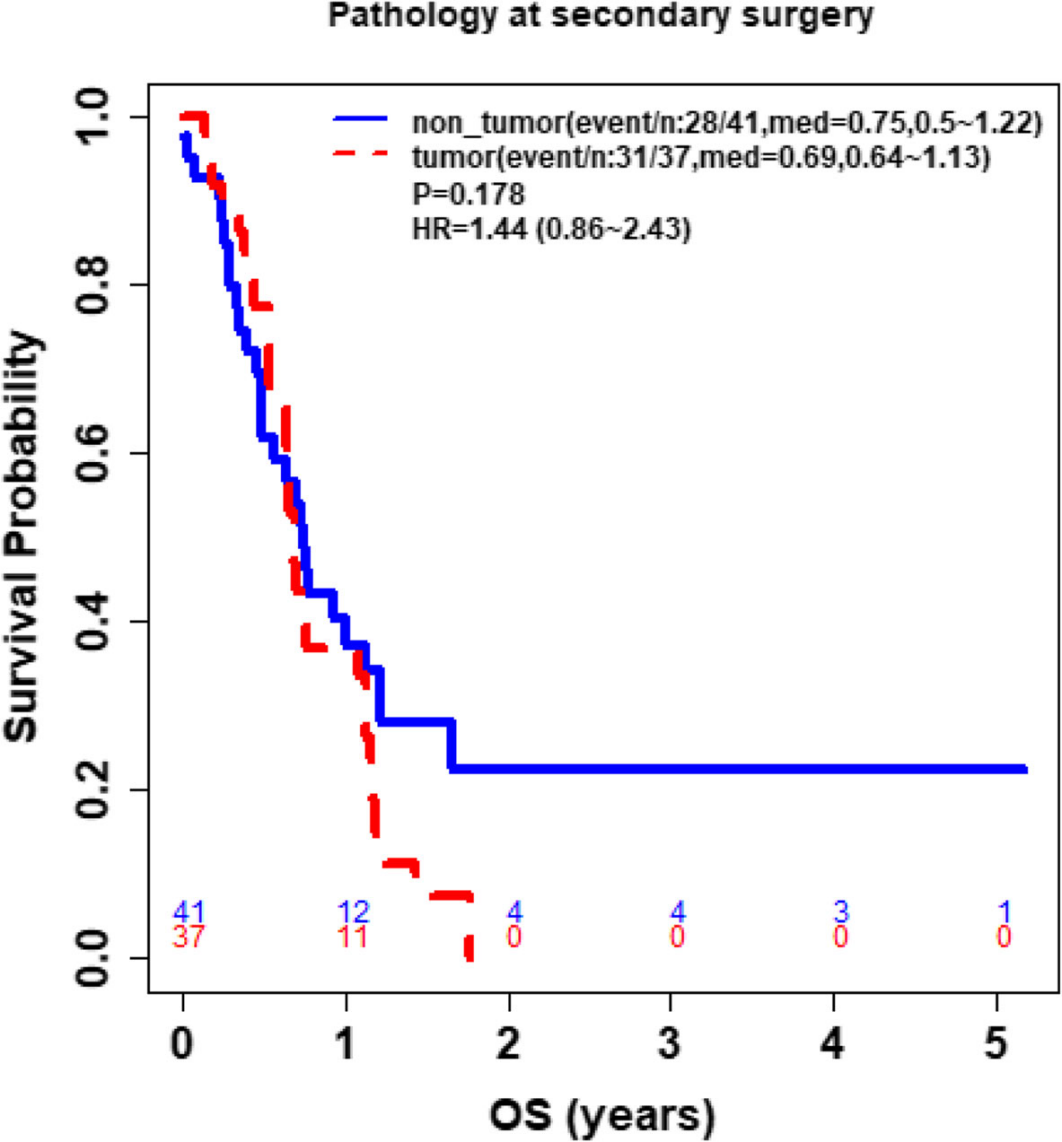
Comparison of overall survival between patients with tumor progression and PsP

**Fig. 8:**
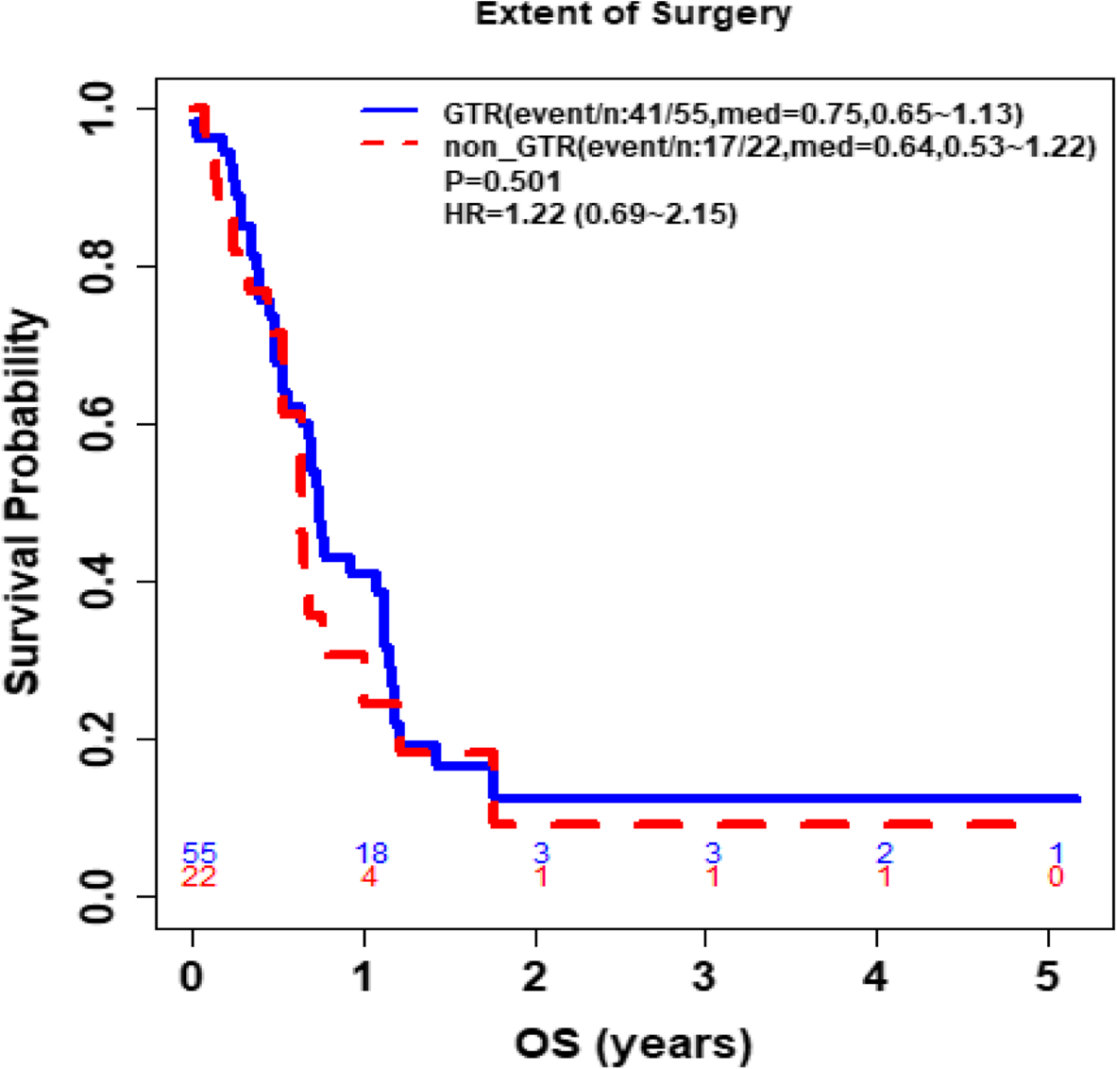
Comparison of overall survival between patients who received GTR versus those who received either Subtotal resection or Biopsy

**Fig. 9:**
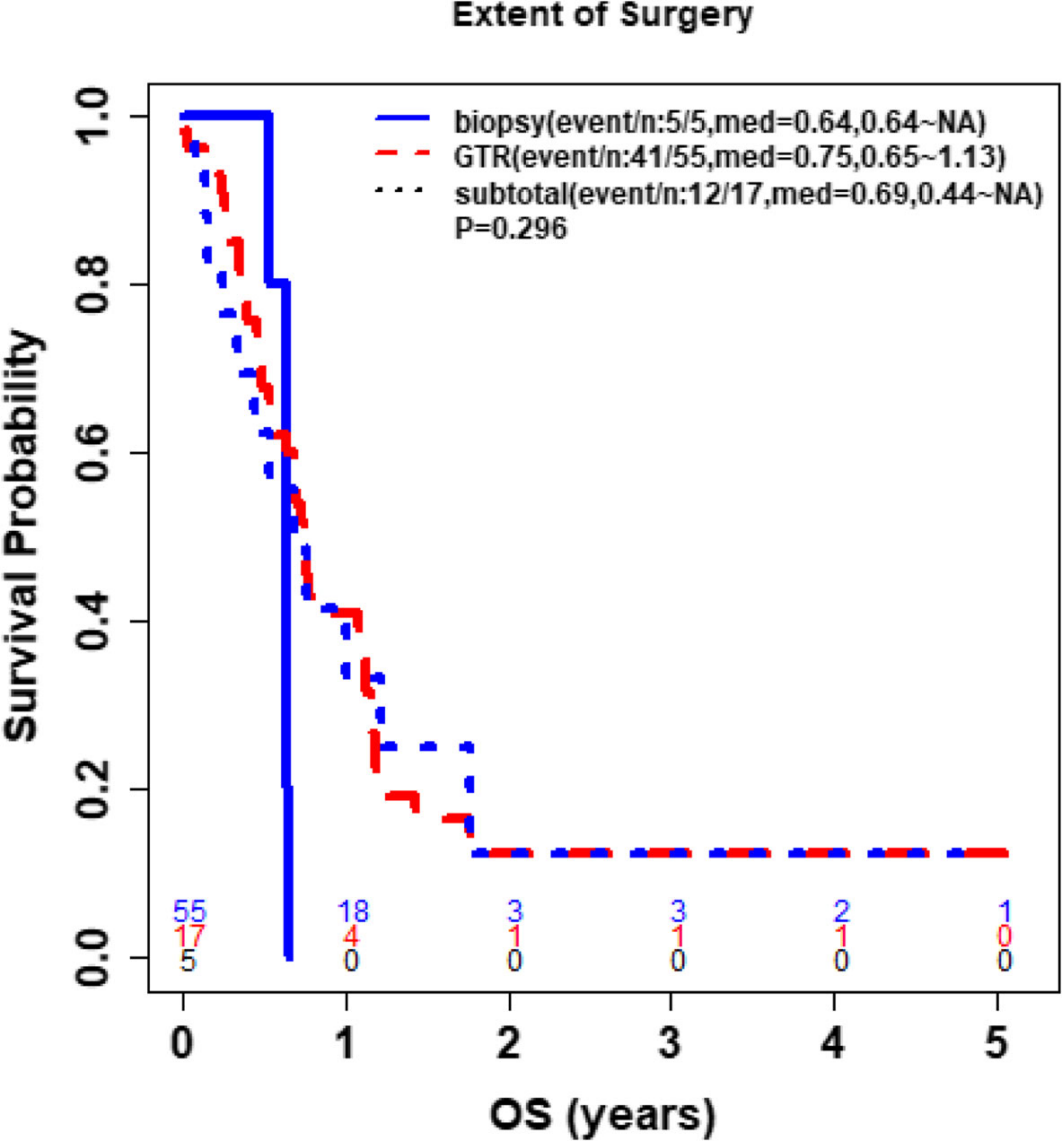
Comparison of overall survival between patients who received GTR versus those who received a Subtotal resection and Biopsy

**Fig. 10:**
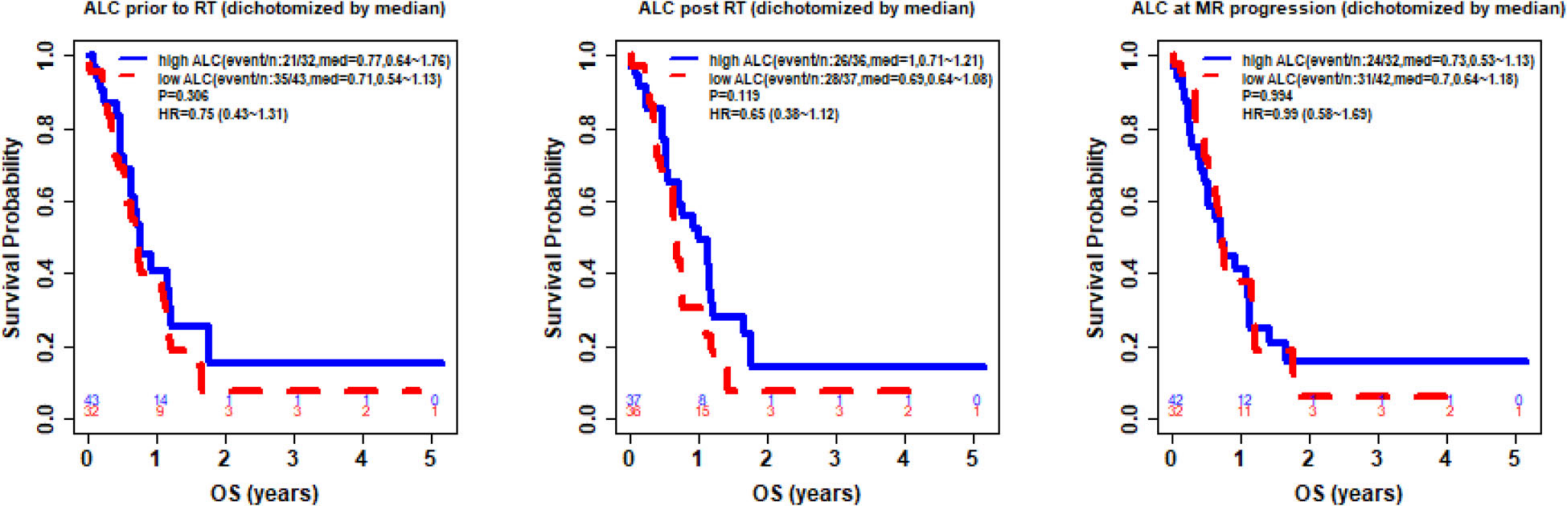
Kaplan Meier Curve of binary ALC (dichotomized by median with number at risk)

Furthermore, study analyses demonstrated that CD4 and ALC at the same time point were highly correlated (**See** Fig. 3), based on the available CD4 counts reported in approximately 20% of the patients. Percentage (%) changes in ALC post RT and at progression from prior to RT were not significantly different between patients with true progression and PsP (**See** Fig. 4**)**. ROC analysis of ALC at each time point and % change in ALC post RT and at progression from prior to RT demonstrated that within this study, ALC did not have predictive value in the differentiation between true tumor progression and PsP (**See** Figs. 5 and 6). Although not statistically significant, study survival analysis demonstrated that patients with true progression had worse OS compared to those who had pseudoprogression (Hazard ratio = 1.44, 95% CI 0.86–2.43, log rank test *P* = 0.178) (**See** Figs. 7, 8 and 9). Although survival analysis results showed that patients with higher ALC (dichotomized by median) appear to have longer OS, these results were not statistically significant. Therefore, in this study cohort, ALC was not prognostic of OS at any treatment time point including before RT/TMZ, after RT/TMZ and at time of MRI documented progression (**See** Fig. 10).

## Discussion

Evaluating treatment of GBMs using MRI remains a problematic task. Although recent adoption of utilizing machine learning algorithm with MRI imaging is gaining increased attention in the field, the majority of current studies have been retrospective in nature, with small sample sizes, without second biopsy pathology confirmation, and require further validation [20, 21]. PsP presents a major challenge in GBM treatment either by leading to unnecessary treatment due to mistaken disease progression, or by delaying therapy when the true disease progression fails to be recognized. In a recent meta-analysis including 73 studies, 36% of MRI demonstrated progression were PsP [22]. Despite increasing research endeavors to work out easily accessible serum biomarkers that may differentiate PsP from true tumor progression in post-treatment GBM patients, the vast majority of study results have been conflicting, and none of the biomarkers studied have been validated [17].

Prior research has suggested that high percentages of CD4+ and CD8+ TILs are correlated with the presence of PsP [18]. Another study investigated the predictive value of Neutrophil to Lymphocyte Ratio (NLR), Lymphocyte to Monocyte Ratio (LMR) as well as Platelet to Lymphocyte Ratio (PLR) to patient survival in newly diagnosed GBM patients. This study found increased LMR (> 1.88) was associated with worse OS. No association between LMR and NLR with survival was reported [23]. ALC, as a sensitive and accurate metric for measuring the number of CD4 cells [24], has never been studied to explore its value as a predictor of PsP versus true tumor progression in GBM patients. Therefore, our study compared ALCs between two cohorts (true progression vs. PsP) at different treatment points hoping to identify a predictive pattern. Unfortunately, this study found no statistically significant differences between the groups, a limitation owed greatly to the small sample size. Study results demonstrated a trend that suggested that at the post-treatment and MRI documented-progression time points, median ALC was consistently lower in true progression group as compared to PsP group.

Relatedly, a previous study found that clinical outcomes of glioblastoma are positively correlated with the number of CD4+ and CD8+ T cells [18]. In addition, CD4 counts have also been used to monitor immunosuppression due to temozolomide therapy in glioma patients [6]. These findings prompted this study’s investigation into the correlation between CD4 counts and ALC, as well as ALC’s predictive value of PsP. Although CD4 counts were missing for many patients, our analysis of the available data showed a strong positive correlation between the CD4 count and ALC. These results support the plausibility of using ALC as a surrogate marker for CD4 counts, pending further large sample size validation. Conversely, a few other studies suggested that ALC values may not be correlated with the presence of TILs [25, 26]. This could explain why our efforts to demonstrate that ALC can be utilized as a serum biomarker to predict the PsP did not reveal a significant association between ALC and PsP, although we did find that ALC dropped significantly after the chemoradiation and this immunosuppression lasted until the first MRI documented progression.

Study analyses also revealed that for those who had PsP, the overall survival was non-significantly better than those who had the true progression, and ALC level at baseline in both groups did not seem to be correlated with overall survival. Recent studies have made great efforts to conquer this clinical challenge outside of imaging modalities. One study suggested that HOX Transcript Antisense Intergenic RNA (HOTAIR), a long noncoding RNAs (lncRNAs) that is overexpressed in GBM and controls GBM cell proliferation, may play a critical role in multiple cancers and may serve as a potential biomarker [27, 28]. Another study suggested that serum HOTAIR has good diagnostic value as a GBM biomarker, and the level of HOTAIR are positively correlated in tumors and serum isolated from GBM patients [29]. Exosomes isolated from peripheral blood as well as cerebrospinal fluid may also be a potential biomarker to help predict true GBM progression [30].

The advantage of this study is that 97% of patients in this study had received standard treatment course with concurrent chemoradiation followed by adjuvant chemotherapy, with only a small percentage (< 5%) of patients having received prior chemotherapy or radiation therapy. This constitutes a great representation of treatment courses patient receive in the real world. Also, all patients with MRI-documented progression had undergone a second biopsy to confirm or rule out tumor progression. This biopsy/pathology confirmation is extremely important but is not always feasible in reality. Our study also has a number of limitations, including its retrospective nature and limited sample size. The small sample size was largely due to a small number of patients who had undergone re-resection after MRI-demonstrated tumor progression. This further emphasizes the importance of identifying easily accessible serum biomarkers to facilitate the differentiation of PsP from true tumor progression, given unreliable MRI findings and the logistical challenges and risks related to re-resection.

Although the mechanism of developing PsP is not clear, PsP following both standard treatment and immunotherapy is thought to be possibly secondary to an immune-mediated response [31]. In the era of immunotherapy, we still believe that immune-related biomarkers may play a role not only in prognosticating survival but also in predicting the treatment response. Therefore, we believe a larger sample size with concurrent investigation of other potential biomarkers such as NLR, MLR, CD4 count, and TILs may yield meaningful findings.

## Conclusion

To our knowledge, this is the first retrospective study that examined the predictive value of ALC in differentiating PsP from true tumor progression in GBM patients who demonstrated disease progression on MRI with pathological tissue confirmation. This study found that ALC dropped significantly after treatment for GBM in both the PsP group and those with true tumor progression. This immunosuppressive effect persisted until MRI documented progression. ALC and percent ALC changes from the baseline to post-treatment or MRI-documented progression were not proven to have predictive value in differentiating PsP from true tumor progression. Those who had true tumor progression had non-significantly worse overall survival than those who had PsP. Future studies with larger sample size are warranted to find easily accessible serum biomarkers to serve the purpose of predicting PsP in post-treatment GBM patients with MRI documented progression.

## Data Availability

All data and material are available upon request.

## Abbreviations

ALC: Absolute lymphocyte count
CBC: Complete blood count
CNS: Central venous system
CTC: Circulating tumor cell
GBM: Glioblastoma multiforme
HGG: High-grade glioma
HNSCC: Head and neck cancer
HOTAIR: HOX Transcript Antisense Intergenic RNA
HR: Hazard ratio
IRB: Institutional review board
LMR: Lymphocyte to monocyte ratio
LncRNA: Long non-coding RNA
MGMT: O^6^-methylguanine–DNA methyltransferase
MRI: Magnetic resonance imaging
NLR: Neutrophil to lymphocyte ratio
NSCLC: Non-small cell lung cancer
OS: Overall survival
PET: Positron emission tomography
PsP: Pseudoprogression
RT: Radiation therapy
TIL: Tumor-infiltrating lymphocyte
TMZ: Temozolomide
TRL: Treatment-related lymphopenia

## References

[1] Louis DN (2016). The 2016 World Health Organization Classification of Tumors of the Central Nervous System: a summary. Acta Neuropathologica.

[2] Mesfin FB, Al-Dhahir MA (2019). Cancer*,* Brain Gliomas. StatPearls Publishing.

[3] Ostrom QT, Gittleman H, Farah P, Ondracek A, Chen Y, Wolinsky Y, Stroup NE, Kruchko C, Barnholtz-Sloan JS. CBTRUS statistical report: Primary brain and central nervous system tumors diagnosed in the United States in 2006-2010. Neuro Oncol. 2013;15 Suppl 2(Suppl 2):ii1-56. 10.1093/neuonc/not151. Erratum in: Neuro Oncol. 2014 May;16(5):760. 10.1093/neuonc/not151 PMC3798196 24137015

[4] Grossman SA (2015). Survival in patients with severe Lymphopenia following treatment with radiation and chemotherapy for newly diagnosed solid tumors. J Natl Compr Cancer Netw.

[5] Yovino S, Grossman SA (2012). Severity, etiology and possible consequences of treatment-related lymphopenia in patients with newly diagnosed high-grade gliomas. CNS Oncol.

[6] Grossman SA (2011). Cancer therapy: clinical immunosuppression in patients with high-grade Gliomas treated with radiation and Temozolomide.

[7] Mendez JS, Govindan A, Leong J, Gao F, Huang J, Campian JL (2016). Association between treatment-related lymphopenia and overall survival in elderly patients with newly diagnosed glioblastoma. J Neuro-Oncol.

[8] Yovino S, Kleinberg L, Grossman SA, Narayanan M, Ford E (2013). The etiology of treatment-related Lymphopenia in patients with malignant Gliomas: modeling radiation dose to circulating lymphocytes explains clinical observations and suggests methods of modifying the impact of radiation on immune cells. Cancer Investig.

[9] Balmanoukian A, Ye X, Herman J, Laheru D, Grossman SA (2012). The association between treatment-related Lymphopenia and survival in newly diagnosed patients with resected adenocarcinoma of the pancreas. Cancer Investig.

[10] Wild AT (2015). The association between Chemoradiation-related Lymphopenia and clinical outcomes in patients with locally advanced pancreatic adenocarcinoma. Am J Clin Oncol.

[11] Campian JL, Sarai G, Ye X, Marur S, Grossman SA (2014). Association between severe treatment-related lymphopenia and progression-free survival in patients with newly diagnosed squamous cell head and neck cancer. Head Neck.

[12] Campian JL, Ye X, Brock M, Grossman SA (2013). Treatment-related Lymphopenia in patients with stage III non-small-cell lung Cancer. Cancer Investig.

[13] Hygino da Cruz LC, Rodriguez I, Domingues RC, Gasparetto EL, Sorensen AG (2011). Pseudoprogression and pseudoresponse: imaging challenges in the assessment of posttreatment glioma. AJNR Am J Neuroradiol.

[14] Parvez K, Parvez A, Zadeh G (2014). The diagnosis and treatment of pseudoprogression, radiation necrosis and brain tumor recurrence. Int J Mol Sci.

[15] Ellingson BM, Chung C, Pope WB, Boxerman JL, Kaufmann TJ. Pseudoprogression, radionecrosis, inflammation or true tumor progression? challenges associated with glioblastoma response assessment in an evolving therapeutic landscape. J Neurooncol. 2017;134(3):495-504. 10.1007/s11060-017-2375-2. Epub 2017 Apr 5. 10.1007/s11060-017-2375-2 PMC7893814 28382534

[16] Hoefnagels FWA (2009). Radiological progression of cerebral metastases after radiosurgery: assessment of perfusion MRI for differentiating between necrosis and recurrence. J Neurol.

[17] Raza IJ, Tingate CA, Gkolia P, Romero L, Tee JW, Hunn MK (2020). Blood biomarkers of Glioma in response assessment including Pseudoprogression and other treatment effects: a systematic review. Front Oncol.

[18] Han S (2014). Tumour-infiltrating CD4(+) and CD8(+) lymphocytes as predictors of clinical outcome in glioma. Br J Cancer.

[19] Campian JL (2015). Pre-radiation lymphocyte harvesting and post-radiation reinfusion in patients with newly diagnosed high grade gliomas. J Neuro-Oncol.

[20] Jang BS, Jeon SH, Kim IH, Kim IA. Prediction of Pseudoprogression versus Progression using Machine Learning Algorithm in Glioblastoma. Sci Rep. 2018;8(1):12516. Published 2018 Aug 21. 10.1038/s41598-018-31007-2. 10.1038/s41598-018-31007-2 PMC6104063 30131513

[21] Ismail M (2018). Shape features of the lesion habitat to differentiate brain tumor progression from pseudoprogression on routine multiparametric MRI: a multisite study. Am J Neuroradiol.

[22] Abbasi AW, Westerlaan HE, Holtman GA, Aden KM, van Laar PJ, van der Hoorn A (2018). Incidence of tumour progression and Pseudoprogression in high-grade Gliomas: a systematic review and meta-analysis. Clin Neuroradiol.

[23] Trombetta L (2017). Correlation Between Inflammatory Markers and Outcome in Patients With Newly Diagnosed Glioblastoma. Int J Radiat Oncol.

[24] Sreenivasan S, Dasegowda V (2011). Comparing absolute lymphocyte count to total lymphocyte count, as a CD4 T cell surrogate, to initiate antiretroviral therapy. J Glob Infect Dis.

[25] Milne K (2012). Absolute lymphocyte count is associated with survival in ovarian cancer independent of tumor-infiltrating lymphocytes. J Transl Med.

[26] Lim A, Coppola D, Chang YD, Anaya DA, Kim DW, Kim RD (2018). Relationship between tumor-infiltrating lymphocytes (TIL) and absolute lymphocyte count (ALC) or lymphocyte to neutrophil ratio (LTN) in cholangiocarcinoma (CCA). J Clin Oncol.

[27] Cantile M (2017). HOTAIR role in melanoma progression and its identification in the blood of patients with advanced disease. J Cell Physiol.

[28] Wang W (2017). Serum HOTAIR as a novel diagnostic biomarker for esophageal squamous cell carcinoma. Mol Cancer.

[29] Tan SK (2018). Serum long noncoding RNA HOTAIR as a novel diagnostic and prognostic biomarker in glioblastoma multiforme. Mol Cancer.

[30] Jiang Y, Qian J, Yang J, Yan X, Xue X, Chang Q (2018). Advances in exosome-related biomarkers for glioblastoma: Basic research and clinical application. Glioma.

[31] Huang RY, Neagu MR, Reardon DA, Wen PY (2015). Pitfalls in the neuroimaging of glioblastoma in the era of antiangiogenic and immuno/targeted therapy - detecting illusive disease, defining response. Front Neurol.

